# Musculoskeletal Biomaterials: Stimulated and Synergized with Low Intensity Pulsed Ultrasound

**DOI:** 10.3390/jfb14100504

**Published:** 2023-10-09

**Authors:** Wanru Jia, Zifei Zhou, Weiwei Zhan

**Affiliations:** 1 Department of Ultrasound, Ruijin Hospital, Shanghai Jiao Tong University School of Medicine, Shanghai 200025, China; jiawanru@126.com; 2 Department of Orthopedics, Shanghai Tenth People’s Hospital, Tongji University School of Medicine, Shanghai 200072, China

**Keywords:** low intensity pulsed ultrasound, bone repair, musculoskeletal biomaterials, tissue engineering, regenerative medicine

## Abstract

Clinical biophysical stimulating strategies, which have significant effects on improving the function of organs or treating diseases by causing the salutary response of body, have shown many advantages, such as non-invasiveness, few side effects, and controllable treatment process. As a critical technique for stimulation, the low intensity pulsed ultrasound (LIPUS) has been explored in regulating osteogenesis, which has presented great promise in bone repair by delivering a combined effect with biomaterials. This review summarizes the musculoskeletal biomaterials that can be synergized with LIPUS for enhanced biomedical application, including bone regeneration, spinal fusion, osteonecrosis/osteolysis, cartilage repair, and nerve regeneration. Different types of biomaterials are categorized for summary and evaluation. In each subtype, the verified biological mechanisms are listed in a table or graphs to prove how LIPUS was effective in improving musculoskeletal tissue regeneration. Meanwhile, the acoustic excitation parameters of LIPUS that were promising to be effective for further musculoskeletal tissue engineering are discussed, as well as their limitations and some perspectives for future research. Overall, coupled with biomimetic scaffolds and platforms, LIPUS may be a powerful therapeutic approach to accelerate musculoskeletal tissue repair and even in other regenerative medicine applications.

## 1. Introduction

Designers of musculoskeletal materials commonly try to recreate hierarchical structures or to offer bone components such as bioactive proteins, minerals, and cells to facilitate and assist the formation of new bone tissue and restore its function [1,2,3]. In addition, the typical fracture healing process inspires novel approaches for bone tissue repair. The majority of bone scaffolds are fabricated by bioceramic, polymer, metal, or hybrid, which typically serve as a mechanical support and a 3D environment for cell adhesion, proliferation, and ingrowth [2,4,5,6]. Musculoskeletal biomaterials include not only transitional bone tissue engineering scaffolds for bone healing, but also biomaterials for spine fusion, bone–tendon, bone–ligament, and cartilage repair. Notably, neurogenesis and neovascularization are also crucial factors which indirectly benefit bone repair [7,8,9,10].

Although it is of potential value to incorporate biological components into bone tissue engineering strategies, the costs of such components limit their further application. Material-only approaches, which exclude biological components and instead rely on the body’s own cells to promote bone regeneration, are highly beneficial for preclinical research and clinical translation [2]. Biophysical stimulating techniques, such as low intensity pulsed ultrasound (LIPUS) stimulation, have shown promise by delivering a combined effect with bioactive materials at a lower cost and a shorter cycle time [11,12]. Generally, LIPUS is a specific type of ultrasound with a frequency of 0.045–3 MHz and intensity of 0.02–l W/cm^2^ [13,14,15,16,17]. The widely used parameters are 1.5 MHz frequency, 30 mW/cm^2^ intensity, 200 μs pulse duration, and 20 min/d exposure time [15,18]. LIPUS has been proven to promote cellular viability, proliferation, differentiation, and migration [13,19,20]. Moreover, LIPUS has shown favorable outcomes in promoting bone fracture healing by inducing molecular, biological, and biomechanical alterations in the fracture vicinity. In addition, LIPUS has been scientifically validated to accelerate bone regeneration in cases of fresh fractures, delayed unions, non-unions, distraction osteogenesis, and musculoskeletal soft tissue injuries [17,18,21,22,23,24,25,26].

According to a meta-analysis of randomized clinical trials, LIPUS therapy may shorten the overall treatment period (mean difference = −15.236 d/cm, 95% confidence interval = −19.902 to −10.569 d/cm) for tibial distraction osteogenesis [27]. Several studies have been conducted in order to improve the growth of osteogenic cells utilizing LIPUS and osteoconductive materials [11,28,29,30]. In certain studies, LIPUS has been shown to improve bone growth and local blood flow in an animal model of fracture repair [31,32]. Meanwhile, it could also contribute to cell spreading, either seeding cells into the scaffold system before implantation or recruiting from graft-surrounded native tissue after implantation in vivo [33,34]. These findings accelerate the development of a clinically applicable LIPUS therapy for bone defects, allowing for the transdermal application of mechanical stress to bone defects without physically destabilizing the defect site.

The mechanisms of ultrasound (US)-responsive nanomaterials include cavitation, acoustic radiation force, acoustic droplet vaporization, hyperthermia, and free radical generation. At least one of these mechanisms can be employed by the nanomaterials [35,36,37,38,39,40]. Acoustic cavitation refers to formation of gas bubbles, pockets, caused by interaction between ultrasonicated materials with acoustic waves [39,41]. Acoustic radiation force is defined as a mechanical force generated by transferring momentum from the ultrasound wave to the medium [38]. Any particles suspended in the fluid will drift, form clusters, and attract or repel one another due to the radiation force [35]. Acoustic droplet vaporization is a process that converts superheated liquid droplets of micron-sized to gaseous microbubbles 5–6 times larger [37]. When focused US beams are targeted at certain tissue, local hyperthermia will occur by absorbing the acoustic energy [40]. Moreover, when US interacts with certain components in a water-based medium, free radical molecules are created for both therapeutic and diagnostic purposes [36,42]. In summary, all these mechanisms may lead to either positive or negative impacts on living tissues.

**Figure 1:**
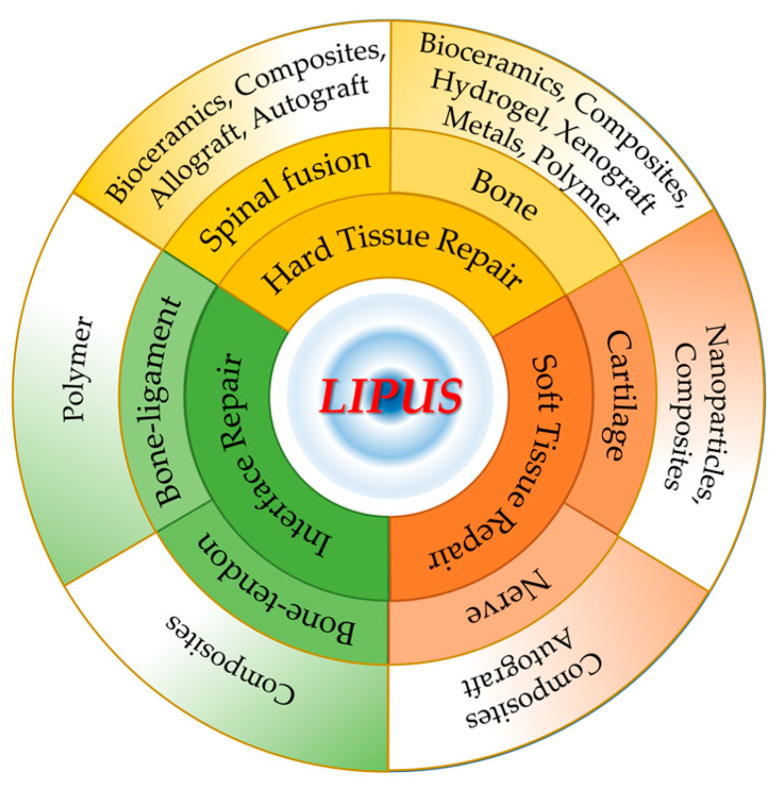
The application of different biomaterials in hard tissue, soft tissue, and interface repair synergized with LIPUS.

In this review, an overview of the enhanced or synergistic effects of LIPUS with musculoskeletal biomaterials will be provided. First, the materials that are designed to promote LIPUS-enhanced regeneration for musculoskeletal injuries are described, including bone, spinal fusion, cartilage, bone–tendon, bone–ligament, and nerve (Figure 1). Furthermore, the most promising LIPUS stimulation techniques will be discussed, including their conducting parameters during stimulus delivery. Finally, a new perspective for LIPUS application in musculoskeletal tissue engineering in the future will be proposed.

## 2. Bone Regeneration

Many researchers focused on the investigation of the effects of LIPUS on bone healing. Osteo-inductive biomaterials are prone to promoting bone regeneration when coupled and modulated with LIPUS appropriately. In this part, we discuss the combined effect of LIPUS with several kinds of biomaterials in promoting bone repair (Table 1).

### 2.1. Bioceramics

Bioceramic materials are intriguing for the fabrication of bone scaffold, since they are desirable alternatives to autogenous or heterogeneous bone grafts. Bioceramics-based scaffolds are good for osteoinduction, osteoconduction, osseointegration, and vascularization [43,44,45,46,47]. In a 1.5 cm rabbit ulna defect model, 20 min of LIPUS stimulation with β-tricalcium phosphate (β-TCP) bone graft increased bone formation at 4 and 12 weeks. Statistically significant differences were found in bone mineral density at 4 weeks, and in new woven bone formation at 4 and 12 weeks. VEGF expression was increased with LIPUS treatment at 4 weeks and remained elevated at 12 weeks compared with controls, while RUNX2 expression levels were elevated with LIPUS treatment at both time points [48]. Wang et al. tried to study the concordant effect of cell/scaffold and LIPUS. Rat bone mesenchymal stem cells (BMSCs) were co-cultured with β-TCP for 2 weeks to form a composite, and then such composite was subcutaneously implanted into rats for further LIPUS treatment [49]. Tests of harvested composites at 5, 10, 25, 50 days showed elevated compressive strength, increased numbers of the vessels, and upregulated expression of CD31 and OCN, denoting that LIPUS stimulation could promote osteogenesis and angiogenesis in the rat BMSCs/TCP composites [49].

The application of LIPUS did not weaken the mechanical property of porous ceramic in vitro. In vivo rabbit experiments revealed that LIPUS treatment for 2 weeks significantly increased osteoblast numbers and bone area, while LIPUS for 3 weeks significantly increased mineralized tissue volume and mineral content in the porous HA ceramic. LIPUS application increased cell migration of MC3T3-E1. It may be a good choice to fill large bone defects in a preclinical model by combining a porous inorganic scaffold with LIPUS [50].

Nagasaki et al. tried to investigate the potential synergistic effects of LIPUS and nanohydroxyapatite in the osteogenic differentiation of human adipose-derived stem cells (hADSCs) [51]. ADSCs isolated from human extirpated buccal fat pad (BFP) were mixed with porcine atelocollagen, with or without nanohydroxyapatite. Then, the mixture was transplanted into the bone defects area of mice calvarium. Experiments in vitro and in vivo revealed the combinational effects of LIPUS and nanocrystalline hydroxyapatite (nHA) in inducing the osteogenic differentiation of ADSCs into osteoblasts, and bone regeneration. The new bone formation only occurred in the defect margin, which can be explained by the heterogeneous sources of cells and organic scaffolds; however, this research provided a novel strategy in autologous sources of ADSCs in combined application with LIPUS [51]. Using stereolithography 3D printer, PEGDA-RGDS-nHA scaffolds (polyethylene glycol diacrylate bioinks containing RGDS peptide and nHA) were fabricated, which could greatly promote hBMSC proliferation rate, filopodia growth, ALP activity, and calcium deposition under LIPUS stimulation [52].

LIPUS has been demonstrated to facilitate the cellular ingrowth in a silicon carbide porous ceramic scaffold, and enhance the proliferation and early osteogenic differentiation of MC3T3-E1 cells [53]. Another study used commercial OsteoBone^TM^ scaffold to find the potential osteogenic capacity of dental follicle cells (DFCs) after LIPUS application. The expression of osteoblast gene markers and formation of mineralized nodules and blood vessels of the DFCs/OsteoBone/LIPUS group increased in vivo. However, the subcutaneous transplantation mouse model limited the observation of potential osteogenesis effect in vivo [29].

### 2.2. Metals

It has been demonstrated that more bone formation was induced in a rabbit nasal bone defect model by porous titanium mesh with high density (10 holes/cm^2^) than that with low density (5 holes/cm^2^), and the application of LIPUS with high density titanium mesh induced a significant augmentation of new bone formation than titanium mesh only [54]. Moreover, the osseointegration of titanium implants in the rabbit metaphyseal area occurred earlier and more adequately in the LIPUS-treated group than in the control group [55]. LIPUS could also promote cell proliferation and migration on a pure titanium plate. In a rabbit study, it accelerated blood flow and maturation of type I collagen around titanium screws, and then promoted bone formation [56].

Electron beam melting (EBM)–microarc oxidation (MAO)-modified porous titanium-6aluminum-4vanadium (Ti6Al4V) scaffolds facilitated cellular filopodia/lamellipodia of MG63 cells, indicated good spreading ability. It was found in vitro that cell proliferation, attachment, and osteogenesis differentiation cultured on these scaffolds were also improved by LIPUS [33]. LIPUS also promoted ALP activity and osteocalcin levels of MC3T3-E1 cultured on porous Ti6Al4V alloy scaffolds, with neither inhibited nor stimulated effect on proliferation or attaching. Moreover, bone ingrowth, bone formation, and maturity were also enhanced in a bony defect model of rabbit mandibles [57,58].

**Figure 2:**
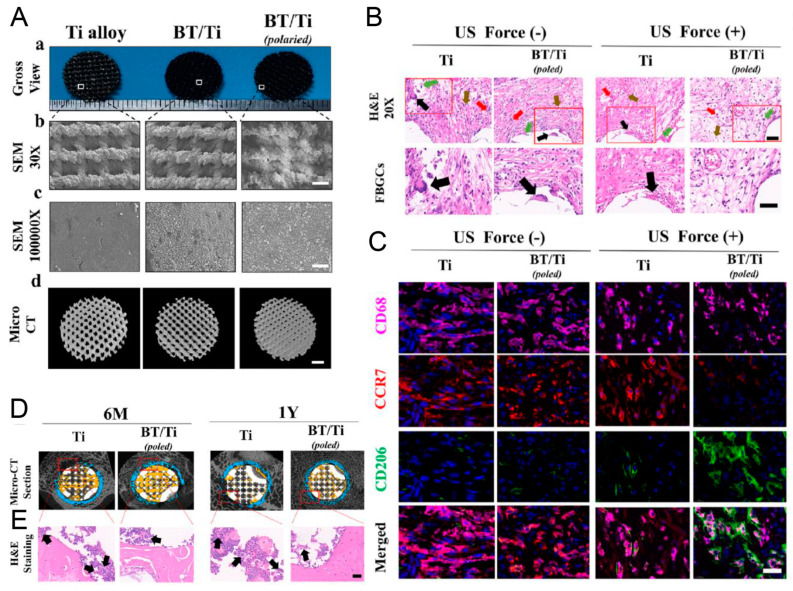
Morphologic views (**A**) and surface observation by SEM (**B**,**C**), and micro-CT 3D reconstruction (**D**,**E**). (**B**) Representative H&E staining images of tissues and FBGCs around scaffolds after subcutaneous implantation for 14 days. (**C**) Representative immunofluorescence images of CD68^+^ macrophages (purple), CCR7^+^ M1 (red), and CD206^+^ M2 (green) macrophages in the tissues after subcutaneous implantation for 14 days. (**D**) Micro-CT section of the osteointegration around the artificial scaffolds. (**E**) Representative H&E staining images of FBGCs in bone tissues around artificial vertebral scaffolds. Reprinted with permission from Ref. [61]. Copyright 2023 Elsevier.

Barium titanate (BaTiO_3_)-coated Ti6Al4V scaffold (BaTiO_3_/Ti6Al4V) improved the surface hydrophilicity and roughness, and showed better cellular attachment, proliferation, and osteoblast differentiation of rabbit BMSCs (rbBMSCs), which could be caused by the LIPUS-triggered piezoelectric effect of BaTiO_3_. Enhanced osteoinduction and osseointegration were found in rabbit radius defects after scaffold implantation and LIPUS application for 6 and 12 weeks [59]. Another study of BaTiO_3_/Ti6Al4V + LIPUS using rat BMSCs (raBMSCs) and sheep femur bone defect model verified such osteogenesis and osseointegration property [60]. However, no experiment was conducted to detect the potential current induced by LIPUS on BaTiO_3_ in these two studies. Another study found that an induced current of 10–17.5 μA was generated by application of LIPUS on BaTiO_3_/Ti6Al4V scaffold. The microcurrent could activate mitochondria, which might be the reason for this piezoelectric effect on cell behaviors, including better viability and adhesion. LIPUS on day 1 caused little damage to cell survival, but the piezodynamic effect weakened the damaged apoptosis and promoted cell proliferation after 4 days’ application. Interestingly, continuous electric cues could be observed even 24 h after intermittent LIPUS stimulation. Thus, there was adequate current to upregulate the expression of osteogenic-related genes [28]. In a subcutaneous implantation rat model, tissues surrounding the poled BaTiO_3_/Ti6Al4V scaffolds showed a high proportion of CD68^+^ CD206^+^ M2 macrophages under LIPUS stimulation. Improvements in macrophage M2 polarization and bone repair were also observed in a sheep cervical corpectomy model. The piezoelectric poled BaTiO_3_/Ti6Al4V scaffold can regulate the immune microenvironment to enhance bone regeneration. This is achieved by inhibiting the inflammatory MAPK/JNK signal pathway and activating oxidative phosphorylation and adenosine triphosphate synthesis in macrophages (Figure 2) [61]. In conclusion, LIPUS might induce electrical signal on electroactive material. Uniform nanosphere-shaped BaTiO_3_ piezoelectric ceramic was coated on the surface of a TC4 titanium alloy to synthesize a BaTiO_3_/TC4 material. Microcurrent (≈10 μA/cm^2^) could be detected when LIPUS was applied on the BaTiO_3_/TC4 disks. Meanwhile, the concentration of intracellular calcium ion and the Ca_V_1.2 protein expression increased. All these mechanisms could introduce synergies of accelerating cell behaviors, cell attachment, migration, proliferation, and osteoblastic differentiation [34].

### 2.3. Sponge- or Hydrogel-Based Composites

Hydrogel or sponge type scaffolds were used as a platform for tissue regeneration. Collagen, especially type I collagen, is the most widely used substrate for 3D cell culture [62,63,64]. Hydrogel or sponge scaffolds made of collagen were widely checked for further application in LIPUS-enhanced bone regeneration [65,66,67]. With the help of atelocollagen sponge, LIPUS alone or simvastatin alone can promote bone regeneration; however, the combination of LIPUS and simvastatin does not induce acceleration in bone formation than LIPUS alone or simvastatin alone [65]. In rat femoral segmental defects, LIPUS enhanced radiographic healing and increased bone volume of rhBMP-2 loaded absorbable collagen sponges. Lower doses (1.2 and 6 μg) of rhBMP-2 delivery induced bone formation, while higher dose (12 μg) induced callus maturation [66]. In addition, LIPUS exposure of seeded hMSCs on magnesium-HA/collagen I hybrid could improve cell colonization and osteogenic differentiation [67].

The treatment of LIPUS showed no influence on cell proliferation within collagen I hydrogel. The elevated gene expression of ALP and osteocalcin denoted that the application of LIPUS could enhance the osteogenetic differentiation of collagen I-encapsulated MC3T3 preosteoblasts. After the BMSCs were localized in type I collagen hydrogels, LIPUS could induce undifferentiated BMSCs to the osteoblastic lineage, and also in vivo fracture healing [68]. Notwithstanding, stiffer collagen I hydrogels could reduce or reverse such osteoblastic response [69]. Either encapsulating cells within a flexible hydrogel or LIPUS exposure could induce high cyclooxygenase 2 (COX-2) and prostaglandin E2 (PGE2) expression. The cumulative higher expression of COX-2 and PGE2 could be observed after combining the two distinct conditions [70].

Moreover, the positive synergistic effect of LIPUS and RGD on the enhancement of proliferation and differentiation of hMSCs was observed. With the hybrid use of LIPUS with RGD, a significant increase was obtained in cell numbers, ALP activity, and mineralized nodule formation assay. With LIPUS, RGD-grafted oxidized sodium alginate/N-succinyl chitosan (RGD–OSA/NSC) hydrogel presented good biological properties in the attachment, proliferation, and osteogenic differentiation of human BMSCs (hBMSCs), suggesting that by combining RGD modification with LIPUS, a high level of bone formation and vascularization would be achieved [71].

### 2.4. GBR/GTR and Xenograft

Guided tissue regeneration (GTR) and guided bone regeneration (GBR) procedures were initially applied to regenerate periodontal tissue, and further used in bone tissue engineering. Generally, GTR and GBR adopted scaffolds or membranes to prevent growth of epithelial and connective tissues into the bone defect, so as to facilitate bone reconstruction. It has been found that LIPUS + collagen barrier membranes can facilitate the osteoblastic differentiation of dog periodontal ligament cells in vitro and promote new alveolar bone formation in vivo [72]. New bone maturation can be accelerated by LIPUS after the implantation of polytetrafluoro ethylene membranes on the surface of a bone defect [73]. The asymmetrically porous membrane was another choice for guided bone regeneration. The cross section of asymmetrically porous polycaprolactone/pluronic F127 membrane showed a column-shaped pore structure. The exterior surface had nanosized pores (≈100 nm) to prevent the infiltration of dense connective tissue but benefit from the permeation of nutrients, while the interior surface had microsized pores (≈100 µm) to improve ingrowth of new bone tissue. The selective permeability, hydrophilicity, and osteoconductivity allowed this membrane to achieve a favorable induction of osteogenesis [74]. In addition, metal membranes showed better induction of new bone than polymer membranes. In vivo, combined with LIPUS, more new bone was observed in rat calvarium defects with a cover of titanium membranes than in those with a cover of GC membrane [75]. LIPUS promoted the repair of periodontal bone defects in beagle dogs, where the bone defect was transplanted with Bio Gide^®^ collagen membrane + autogenous bone graft [76].

Before implantation of a cell-seeded scaffold, pre-treatment of such scaffold with LIPUS could probably facilitate cell ingrowth and thus accelerate fracture healing and tissue regeneration. For MC3T3 cells in 3D trabecular bone scaffold, LIPUS treatment yielded enhanced calcific deposition, but reduced proliferation [77].

### 2.5. Polymers or Microbubbles

**Figure 3:**
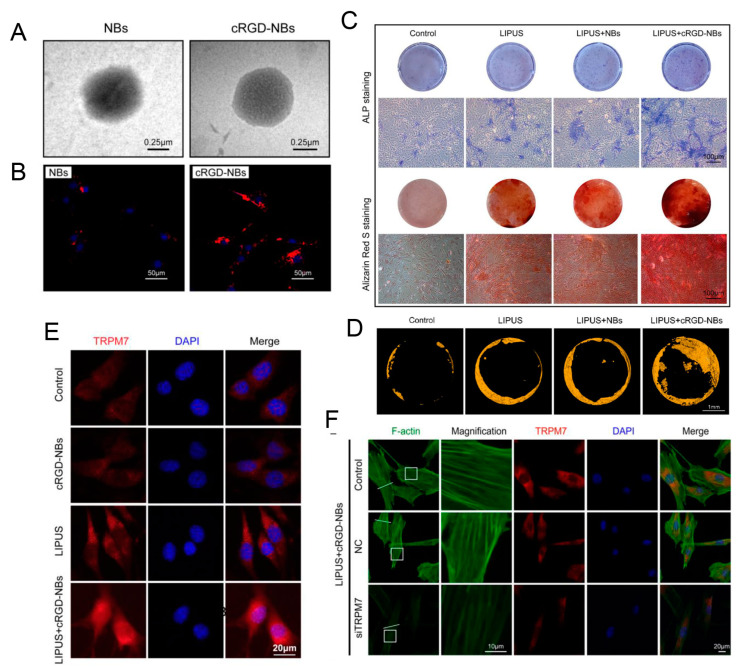
(**A**) Representative TEM images of NBs and cRGD-NBs. (**B**) Representative images of BMSCs co-incubated with Dil-labeled NBs and cRGD-NBs. (**C**) Alkaline phosphatase staining on day 7 and Alizarin Red S staining on day 21. (**D**) Micro-CT images of mouse calvarial bone regeneration after 4 weeks of treatment. (**E**) Representative immunofluorescence images of BMSCs co-incubated with cRGD-NBs and LIPUS, TRPM7 (red), and DAPI (blue). (**F**) Representative immunofluorescence images of BMSCs co-incubated with cRGD-NBs and LIPUS treatment for 4 h, TRPM7 (red), F-actin (green), and DAPI (blue). Reprinted from Ref. [78].

The mechanical effects of LIPUS could be amplified by adding microbubbles into cell culture, such as local shear forces and controllable mechanical stress in cells. Yao et al. reported cyclic arginine-glycine-aspartic acid-modified nanobubbles (cRGD-NBs), which could target BMSCs mediated by integrin receptors. LIPUS/cRGD-NBs could promote the osteogenic differentiation of BMSCs induced by polymerization of actin microfilaments, TRPM7 regulation (Figure 3), and extracellular Ca^2+^ influx [78]. Microbubbles coated by a monolayer of lipids have been approved by the FDA for contrast-enhanced ultrasound imaging [79]. Integrating LIPUS and lipid mixture-coated microbubbles was proven to be effective in boosting cell proliferation and osteogenic differentiation of hBMSCs which were cultured on 3D printed porous poly(lactic acid) scaffolds. The microbubbles could maintain stable structure during LIPUS exposure. Sustained oscillations by LIPUS demonstrably contributed to the transmission of ultrasound energy toward surrounding cells of microbubbles [80]. Specific three-dimensional architectures of 3D-printed scaffolds are good for the acoustic wave transmission of LIPUS. Cells attached on the 3D-printed scaffolds receive more sonic stimulation, and thus tend to exhibit active cellular activity.

Huang demonstrated the ability of poly-L-lactic acid (PLLA) electrospun nanofibrous membrane coupled with LIPUS in enhancing the development of nascent bone using the rabbit tibia defect model [81]. Moreover, combining LIPUS with lipid microbubbles on poly (lactic-glycolic acid copolymer) (PLGA)/α-tricalcium phosphate (TCP) 3D-printed scaffolds can also enhance the growth and osteogenesis of BMSCs [82]. Ramie-based carboxymethyl cellulose (CMC) displayed cytocompatity of MC3T3-E1 cells, and synergistic effects caused by LIPUS and CMC further promoted cellular proliferation and osteogenic differentiation [83].

**Table 1:** LIPUS + biomaterials for bone repair.

No	Biomaterials	Constituent	Evidence In Vitro						Evidence In Vivo					Ref.
			Cell	Prolif	Adhes	Migra	Osteog Differ		Animal	Osteo-ind	Osteo-cond	Osseo-int	Angio-ge	
1	Bioceramics	TCP	/	/	/	/	/	/	Rabbit	+	/	/	+	[48]
2	Bioceramics	Hydroxyapatite	MC3T3-E1	/	/	+	+	/	Rabbit	+	+	/	/	[50]
3	Metals	Titanium	/	/	/	/	/	/	Rabbit	/	/	+	/	[55]
4	Polymers	ePTFE	/	/	/	/	/	/	Dog	+	/	/	/	[73]
5	Metals	Titanium	/	/	/	/	/	/	Rat	+	/	/	/	[75]
6	Polymers	PCL/F127	/	/	/	/	/	/	Rat	+	+	/	/	[74]
7	Metals	Titanium	MG63	+	+	/	+	/	Rabbit	+	/	+	/	[56]
8	Composites	Bio Gide^®^/autogenous bone graft	/	/	/	/	/	/	Dog	+	/	/	/	[76]
9	Composites	raBMSCs/TCP	/	/	/	/	/	/	Rat	+	/	/	+	[49]
10	Composites	Sim@ACS	/	/	/	/	/	/	Rabbit	+	/	/	/	[65]
11	Hydrogel	RGD–OSA/NSC	hBMSCs	+	+	/	+	Vascul	/	/	/	/	/	[71]
12	Composites	rhBMP-2@ACS	/	/	/	/	/	/	Rat	+	/	/	/	[66]
13	Composites	Atelocollagen/nanohydroxyapatite	hADSCs	/	/	/	+	/	Mouse	+	/	/	/	[51]
14	Bioceramics	Silicon carbide	MC3T3-E1	+	+	+	+	/	/	/	/	/	/	[53]
15	Composites	PEGDA-RGDS-nHA	hBMSCs	+	+	/	+	/	/	/	/	/	/	[52]
16	Hydrogel	Collagen I	MC3T3	-	/	/	+	/	/	/	/	/	/	[69]
17	Metals	Titanium	/	/	/	/	/	/	Rabbit	+	/	/	/	[54]
18	Composites	MgHA/Col I	hMSCs	+	/	/	+	/	/	/	/	/	/	[67]
19	Metals	Ti6Al4V	MC3T3-E1	-	-	/	+	/	Rabbit	+	+	/	/	[57]
20	Polymer	PLLA	/	/	/	/	/	/	Rabbit	+	/	/	/	[81]
21	Bioceramics	OsteoBone^TM^	DFCs	/	+	/	+	/	Mouse	/	/	/	+	[29]
22	Xenograft	Trabecular bone	MC3T3	-	/	/	+	/	/	/	/	/	/	[77]
23	Composites	Collagen	dPDLCS	/	/	/	+	/	Dog	+	/	/	/	[72]
24	Composites	LMBs + PLA	hBMSCs	+	/	/	+	/	/	/	/	/	/	[80]
25	Metals	Ti6Al4V	MG63	+	+	/	+	/	/	/	/	/	/	[33]
26	Metals	Ti6Al4V	MC3T3-E1	-	/	/	+	/	Rabbit	+	+	/	/	[58]
27	Metals	BaTiO_3_/Ti6Al4V	rbBMSCs	+	+	/	+	/	Rabbit	+	/	+	/	[59]
28	Metals	BaTiO_3_/Ti6Al4V	raBMSCs	/	/	/	+	/	Sheep	+	+	+	/	[60]
29	Metals	BaTiO_3_/Ti6Al4V	raBMSCs	+	+	/	+	/	/	/	/	/	/	[28]
30	Metals	BaTiO_3_/TC4	MC3T3-E1	+	+	+	+	/	/	/	/	/	/	[34]
31	Metals	BaTiO_3_/Ti6Al4V	RAW264.7 MC-3T3	/	/	+	+	Polari	Rat Sheep	+	+	+	/	[61]
32	Composites	Carboxymethyl cellulose	MC3T3-E1	+	/	+	+	/	/	/	/	/	/	[83]
33	Composites	cRGD-NBs	mBMSCs	/	/	/	+	/	/	/	/	/	/	[78]
34	Composites	PLGA/TCP	raBMSCs	+	+	/	+	/	/	/	/	/	/	[82]
35	Hydrogel	Collagen I	/	/	/	/	/	/	Rat	+	/	/	/	[68]

Prolif: proliferation, Adhes: adhesion, Migra: migration, Osteog Differ: osteogenic differentiation, Vascul: vascularization, Polari: polarization, Osteoind: osteoinduction, Osteocond: osteoconduction, Osseoint: osseointegration, Angioge: angiogenesis. hBMSCs: human bone mesenchymal stem cells, hADSCs: human adipose-derived stem cells, DFCs: dental follicle cells, dPDLCS: dog periodontal ligament cells, rbBMSCs: rabbit bone mesenchymal stem cells, raBMSCs: rat bone mesenchymal stem cells. TCP: tricalcium phosphate, ePTFE: expanded polytetrafluoro ethylene, PCL/F127: polycaprolactone/pluronic F127, Sim@ACS: simvastatin loaded atelocollagen sponge, RGD: arginine-glycine-aspartic acid, RGD–OSA/NSC: RGD-grafted oxidized sodium alginate/N-succinyl chitosan, rhBMP-2@ACS: rhBMP-2 loaded absorbable collagen sponges, PEGDA: polyethylene (glycol) diacrylate, nHA: nanocrystalline hydroxyapatite, MgHA/Col I: magnesium-HA/collagen I, Ti6Al4V: titanium-6aluminum-4vanadium, PLLA: poly(L-lactic acid), DFBA: demineralized freeze-dried bone allograft, LMBs + PLA: lipid-coated microbubbles + poly(lactic acid) porous scaffolds, cRGD-NBs: cyclic arginine-glycine-aspartic acid-modified nanobubbles, PLGA/TCP: poly(lactic-glycolic acid copolymer)/α-tricalcium phosphate. +: Positive, -: Invalid or negative, /: not tested.

## 3. Spinal Fusion

In addition to the effective promotion of fracture repair, including new fractures, the method may also be of use in delayed union or nonunion and bone defects [84,85,86,87]. Meanwhile, LIPUS therapy may be a useful means to ensure successful spine fusion (Table 2). Several studies focused on spinal fusion using LIPUS and autologous bone graft. LIPUS treatment improved the lumber fusion rate of autologous iliac bone graft after 12 weeks of implantation. The ABG + LIPUS group achieved 100% fusion rate, both in radiographic and histologic fusion, while the ABG group achieved 78% radiographic fusion and 44% histologic fusion [88]. Another study verified the conclusion in a rabbit model of posterolateral intertransverse process spine arthrodesis using muscle-pediculated bone grafts [89]. In a rabbit lumbar posterolateral fusion model, augmented by LIPUS, stiffer fusion mass and an analogous fusion rate can be achieved by laminectomy chip bone graft (LCBG) than those of an AIBG [90]. In nicotine-administrated rabbit, LIPUS could not promote fusion rate without any implantation, and remained at 0% in the control group with no implantation and LIPUS. However, LIPUS could increase the fusion rate from 29% to 57% in the AIBG-implanted rabbits [91].

Hui et al. established a posterior spinal fusion model in New Zealand white rabbit, to evaluate the synergistic effects of LIPUS by implanting porous TCP bioceramic scaffolds and mesenchymal stem cells (MSCs) [92]. They found that LIPUS could enhance endochondral ossification at the fusion site and bone formation with porous TCP scaffold which was impregnated with MSCs. Thus, it was LIPUS that could achieve better osseointegration between the host bone and implanted composites [92]. LIPUS can increase rabbit spinal posterolateral fusion, bone density, trabecular bone formation, and accelerate bone in-growth into hydroxyapatite ceramics [93]. Interestingly, in a rabbit model of posterolateral lumbar fusion, there was no significant difference in the number of chondrocytes and relative gray-scale between the hydroxyapatite and the AIBG [94].

**Figure 4:**
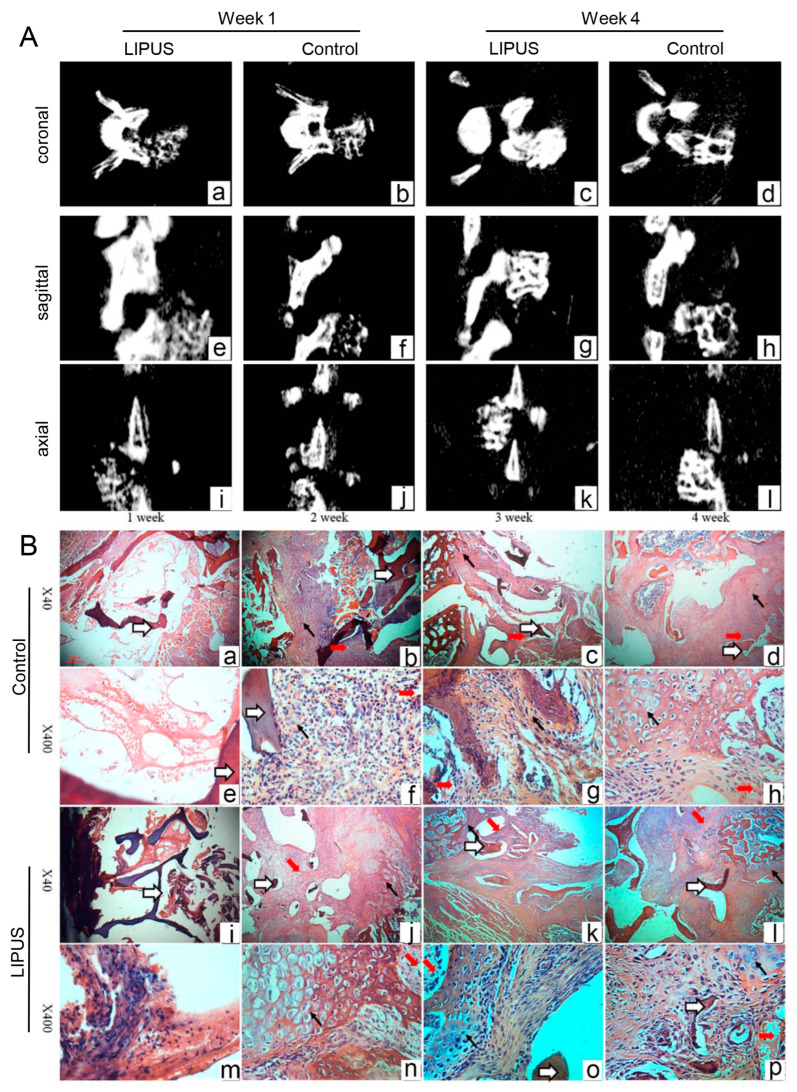
(**A**) Micro-CT scan views at 1 and 4 weeks post-surgery in the LIPUS group and control group. (**B**) H&E photomicrographs of the fusion mass in control and LIPUS-treated rats at 1–4 weeks. White arrows denotes allografts. Fat red arrows denotes microvessels. Thin red arrows denotes chondrocytes. Reprinted from Ref. [95].

Generally, demineralized freeze-dried bone allograft (DFDBA) was usually used for studies on LIPUS-accelerated spinal fusion. Stimulated by LIPUS, a number of type H vessels could be observed in the fusion mass of rat spinal fusion model, and more osteoblasts were located on the bone callus of the allograft and were enclosed by type H vessels (Figure 4) [95]. Further study indicated that LIPUS could promote not only osteoblast differentiation but also cell migration of osteoblast-like MG63 cells, which contributed to DFDBA-induced spinal fusion. The upregulated sonic hedgehog (Shh) signal pathway was involved in those cell behaviors. In contrast, inhibited Shh signaling reduced the migratory and proliferative ability of MG63 cells and impeded the efficacy of LIPUS treatment [96]. Cell experiments of Raw264.7 cells and bone marrow-derived macrophages (BMDM) indicated that the polarization changes of macrophages were found from inflammatory type M1 to resident type M2 after LIPUS application. The authors deemed that the macrophages’ earlier polarization transition might be one cause of the confirmed effect of DFDBA + LIPUS on spinal fusion [97]. Overall, multiple factors, including vessel formation, Shh signaling, and polarization transition, may be involved in the LIPUS-enhanced spinal fusion of DFDBA.

During callus formation or bone remodeling, calcitonin gene-related peptide (CGRP)-positive sensory nerve fibers proliferated rapidly and may play an important role in bone repair [98,99]. However, sensory innervation decreased or even disappeared when the delayed fracture or non-union existed [100,101]. The rhBMP-4-loaded porous poly-D, L-lactic acid blocks were implanted in the rabbit under bilateral posterolateral intertransverse process fusion. After LIPUS treatment, the number and density of CGRP-positive nerve fibers were higher in newly formed cartilage and bone tissue. Thus, LIPUS promoted the growth of CGRP sensory nerves into heterotopic bone, thereby contributing to the promotion of LIPUS on ectopic ossification [102]. Another study from a rat spinal fusion model confirmed that CGRP innervation located closely surrounding the demineralized freeze-dried bone allograft and newly formed cartilage [103].

**Table 2:** LIPUS + biomaterials for spinal fusion.

No	Biomaterials	Constituent	Evidence In Vitro				Evidence In Vivo						Ref.
			Cell	Prolif	Migra	Polari	Animal	CGRP Innerv	Osteo-ind	Osteo-cond	Osseo-int	Angio-ge	
1	Autograft	AIBG	/	/	/	/	Dog	/	+	/	/	/	[88]
2	Autograft	MPBG	/	/	/	/	Rabbit	/	+	/	/	/	[89]
3	Bioceramics	HA	/	/	/	/	Rabbit	/	+	+	/	/	[93]
4	Composites	BMP4/PDLA	/	/	/	/	Rabbit	+	+	/	/	/	[102]
5	Composites	DFDBA	/	/	/	/	Rat	+	+	/	/	/	[103]
6	Bioceramics	TCP	/	/	/	/	Rabbit	/	+	+	+	/	[92]
7	Autograft	LCBG	/	/	/	/	Rabbit	/	+	/	/	/	[90]
8	Autograft	AIBG	/	/	/	/	Rabbit	/	+	/	/	/	[94]
9	Autograft	AIBG	/	/	/	/	Rabbit #	/	+	/	/	/	[91]
10	Allograft	DFDBA	/	/	/	/	Rat	/	+	/	/	+	[95]
11	Allograft	DFDBA	MG63	+	+	/	Rat	/	+	/	/	/	[96]
12	Allograft	DFDBA	Raw264.7, BMDM	/	/	+	Rat	/	+	/	/	/	[97]

Prolif: proliferation, Adhes: adhesion, Migra: migration, Chondr Differ: chondrogenic differentiation, Polari: polarization, Innerv: innervation, Chondrogen: chondrogenesis, Vascul: vascularization, CGRP: calcitonin gene-related peptide, Osteoind: osteoinduction, Osteocond: osteoconduction, Osseoint: osseointegration, Angioge: angiogenesis. AIBG: autologous iliac bone graft, MPBG: muscle-pediculated bone grafts, HA: hydroxyapatite, BMP4/PDLA: BMP4-loaded poly-D,L-lactic acid, DFDBA: demineralized freeze-dried bone allograft, TCP: tricalcium phosphate, LCBG: laminectomy chip bone graft. +: Positive, /: not tested, #: nicotine-administered.

## 4. Osteonecrosis/Osteolysis

Non-medical LIPUS has been used in common musculoskeletal disorders, including osteoporosis, osteonecrosis, and osteolysis [104,105]. By increasing bone formation and decreasing bone resorption, LIPUS was found to counteract the bone loss effects induced by spinal cord injury. The osteogenic effects of LIPUS lay in partially restoring endochondral ossification during callus formation, which would finally result in newly formed tissue with enhanced microarchitecture and mechanical integrity [106].

**Figure 5:**
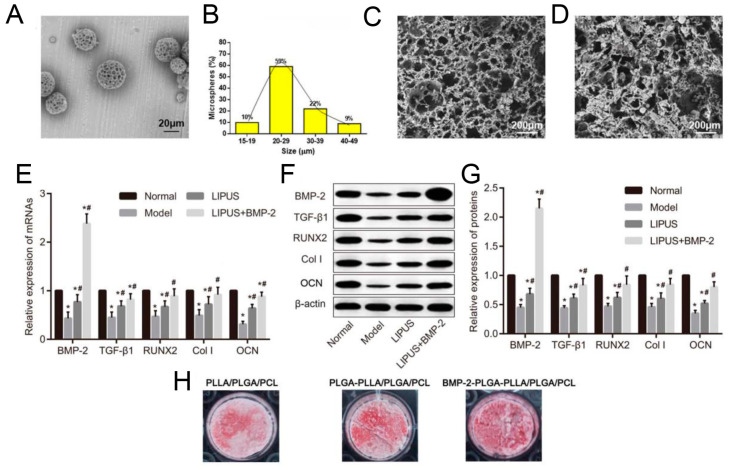
(**A**) TEM images of BMP-2-loaded PLGA microspheres. (**B**) Particle size distribution of BMP-2-loaded PLGA nanoparticles. (**C**) Surface morphology of the PLLA/PLGA/PCL scaffold. (**D**) Morphology of the BMP-2-loaded PLGA-PLLA/PLGA/PCL composite scaffold. (**E**) Measurement of BMP 2, TGF β1, RUNX 2, and Col I and OCN mRNA expression in tissues by RT-PCR. (**F**,**G**) Measurement of BMP 2, TGF β1, RUNX 2, Col I, and OCN protein expression in tissues by Western blotting. * denotes *p* < 0.05 compared with Normal group. # denotes *p* < 0.05 compared with Model group. (**H**) Measurement of calcium deposition by alizarin red staining in MC3T3 E1 cell lines. Reprinted from Ref. [109].

In a steroid-associated osteonecrosis rabbit model, LIPUS was proven to promote bone regeneration by increasing osteogenesis and neovascularization [32]. The potential biomechanical mechanism of LIPUS in the treatment of disuse osteoporosis may be the mechanical micro-environment improvement of trabecular bone and osteoblasts [107]. Pilot studies have been conducted regarding the combined effect of biomaterials and LIPUS (Figure 5) [108,109].

Periprosthetic osteolysis was the leading cause of polyethylene artificial joint invalidation [110,111]. Yan et al. reported that LIPUS could prevent or delay the polyethylene debris-caused osteolysis. The changes of shear strength, bone mineral density (BMD) and histopathology indicated that LIPUS-induced bone growth reversed the polyethylene-caused periprosthetic osteolysis [108], and the underlying mechanism may lie in the stimulation of bone tissue growth and inhibition of fibroblast growth. Further studies are needed to determine whether osteoclasts played an essential role in the LIPUS treatment of periprosthetic osteolysis [108].

Corticosteroid use is one of the major risks of osteonecrotic lesions; LIPUS treatment alone was validated to contribute to the alleviation of osteonecrosis [112,113], so it was a promising strategy to take advantage of the biomaterials-enhanced effect after LIPUS intervention. In another steroid-induced osteonecrosis model, bone morphogenetic protein-2 (BMP-2)-loaded poly-L-lactic acid/polylactic-co-glycolic acid/poly-ε-caprolactone (PLLA/PLGA/PCL) composite scaffolds stimulated by LIPUS could facilitate osteoblast differentiation, vascularization, and bone formation [109].

## 5. Cartilage Repair

Chen et al. designed a four-layer scaffold, including layer 1: for cartilage repair, layer 2: for cartilage calcification, layer 3: for spatial distribution restriction of cells, and layer 4: for bone repair [114]. The hybrid use of multiple growth factors and LIPUS treatment exhibited good potential in facilitating vascularization and osteochondral repair [114] (Table 3).

In rabbit articular cartilage defects, LIPUS promoted the hyaline chondroid tissue formation after transplantation of allogeneic chondrocytes–calcium alginate gel composite (C-CAG). The smooth surface and integration degree optimized such effect in the LIPUS/C-CAG group. In line with the gross appearance, histological observation found that the collagen II positive area in the LIPUS/C-CAG group was larger than that in the model group and C-CAG group [115].

Combined with LIPUS, liposome-encapsulated rapamycin (L-rapa) not only increased proteoglycan production in human normal chondrocytes, but also improved type II collagen production. Moreover, in human osteoarthritis (OA) chondrocytes (HOACs), L-rapa + LIPUS upregulated mRNA expression or synthesis of aggrecan, type II collagen, and proteoglycan, while inhibiting the expression of MMP-13 and IL-6. Immunohistochemical findings from spontaneous OA Dunkin-Hartley guinea pig models proved significant enhancement of glycosaminoglycans and type II collagen in articular cartilage in the L-rapa + LIPUS group. Moreover, decreased expression of MMP-13 was principally consistent with those found in HOACs in vitro. The verified results in vitro and in vivo evidently ascertained that the L-rapa combined with LIPUS showed promising anabolic and anti-catabolic activities against OA [116].

However, one study reported that LIPUS had limited potential in stimulating the synthesis of sulphated glycosaminoglycan from bovine articular chondrocytes which were cultured in monolayer or agarose constructs [117]. Composites of bovine chondrocyte-fibrinogen exhibited no significant difference in neocartilage formation between LIPUS-treated and sham-treated groups [118]. Subcutaneously implanted composites in the backs of nude mice might account for the similar cartilage maturation and regeneration stability.

**Figure 6:**
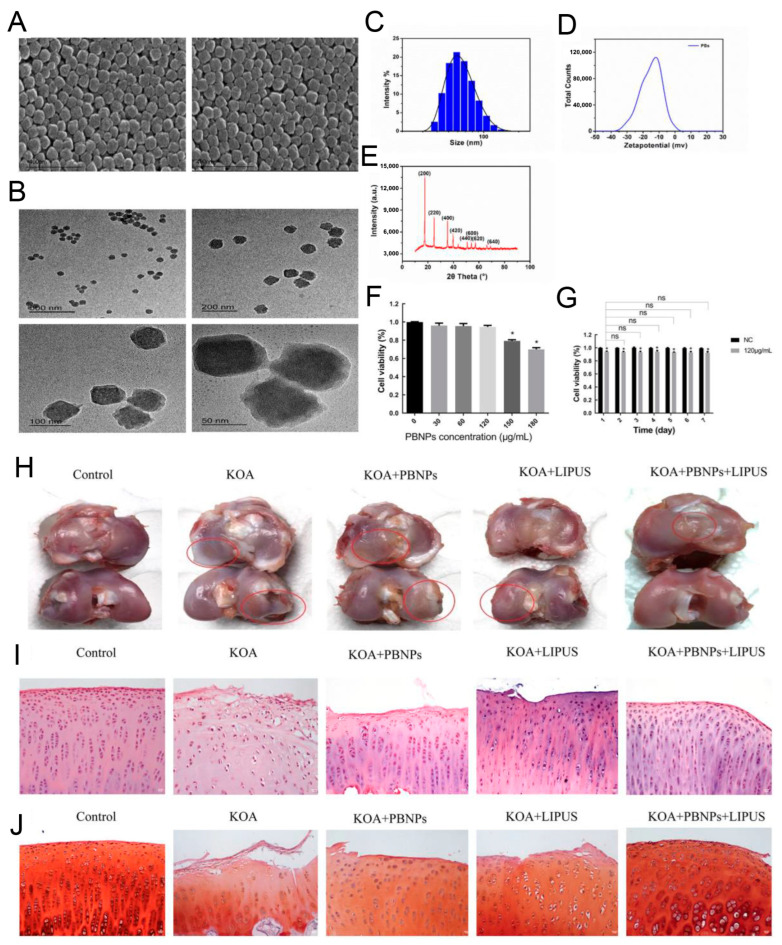
SEM image (**A**), TEM image (**B**), size distribution profiles (**C**), Zeta potential (**D**), and X-ray diffractometer result (**E**) of PBNPs. (**F**) Cell viability of various PBNP concentrations. (**G**) Effect of PBNPs (120 μg/mL) on cells viability for 7 d. Macroscopic observation (**H**) and H&E staining (**I**) and Safranin O staining (**J**) of effects of PBNPs/LIPUS on repair of the femoral condylar cartilage. * *p* < 0.05 vs. 0 μg/mL PBNPs group. ns: no significance. Reprinted from Ref. [119].

The eradication of reactive oxidative stress (ROS) could relieve chondrocyte apoptosis and extracellular matrix (ECM) degradation, which were pathological changes in cartilage suffering from osteoarthritis. Prussian blue nanoparticles (PBNPs) + LIPUS application drastically reversed lipopolysaccharide (LPS)-induced cellular ROS level and apoptosis rate by activating the PI3K/Akt/mTOR pathway. Meanwhile, PBNPs/LIPUS combination treatment resulted in the inhibition of IL-1β and MMPs by the suppression of JNK/c-Jun signal pathway in LPS-incubated chondrocytes. The anterior cruciate ligament was transected to construct a knee osteoarthritis rabbit model. Consistent with the result from chondrocytes, PBNPs + LIPUS application could activate the PI3K/Akt/mTOR signaling and suppress the JNK/c-Jun axis, leading to reverse cellular apoptosis and ECM degradation, which in turn provided an exciting repair of femoral condylar cartilage (Figure 6) [119].

Microbubbles, one kind of clinically approved agent for contrast-enhanced ultrasound imaging, could be combined with LIPUS for further application in bone tissue engineering. Along with LIPUS, lipid-coated, perfluorobutane-filled microbubbles could boost the proliferation properties and chondrogenic differentiation of human mesenchymal stem cells (hMSCs) which were cultured on 3D printed poly-(ethylene glycol)-diacrylate (PEG-DA) hydrogel scaffold (LPM + PEG-DA). The hMSCs produced more glycosaminoglycan (GAG) of 17% and type II collagen of 78% in the LIPUS+ microbubbles + LPM + PEG-DA group, whereas in the LIPUS group, they were 5% and 44% [120].

**Table 3:** LIPUS + biomaterials for chondral/osteochondral repair.

No	Biomaterials	Constituent	Evidence In Vitro			Evidence In Vivo				Ref.
			Cell	Prolif	Chondr Differ	Animal	Chondrogen	Osteo-ind	Angio-ge	
1	Composites	C-CAG	/	/	/	Rabbit	+	/	+	[115]
2	Composites	LPMBs + PEG-DA	hMSCs	+	+	/	/	/	/	[120]
3	Composites	Four-layers scaffold	/	/	/	Rabbit	+	+	+	[114]
4	Composites	L-rapa	Human Chondrocyte	-	+	Pig	+	/	/	[116]
5	Nanoparticles	PBNPs	Rabbit Chondrocyte	#	/	Rabbit	+	/	/	[119]

Prolif: proliferation, Chondr Differ: chondrogenic differentiation, Chondrogen: chondrogenesis, Osteo-ind: osteoinduction, Angio-ge: angiogenesis. hMSCs: human mesenchymal stem cells. C-CAG: chondrocytes-calcium alginate gel, LPMBs + PEG-DA: lipid-coated, perfluorobutane-filled microbubbles and poly-(ethylene glycol)-diacrylate (PEG-DA) hydrogel scaffold, Four-layers scaffold: first layer: hydrogel of oxidized sodium alginate and N-succinyl chitosan (OSA/NSC) loaded with FGF-2, BMP-2, and TGF-1; second layer: hydrogel of OSA/NSC loaded with micro hydroxyapatite (μHA) and wnt/β-catenin; third layer: PCL/PEG electrospun fiber membrane; fourth layer: porous composite of SA/nano HA/BMP-2-loaded coaxial short fibers, L-rapa: liposome-encapsulated rapamycin, PBNPs: Prussian blue nanoparticles. +: Positive, -: Invalid or negative, /: not tested, #: Reverse LPS-induced apoptosis.

## 6. Bone–Ligament or Bone–Tendon Repair

**Figure 7:**
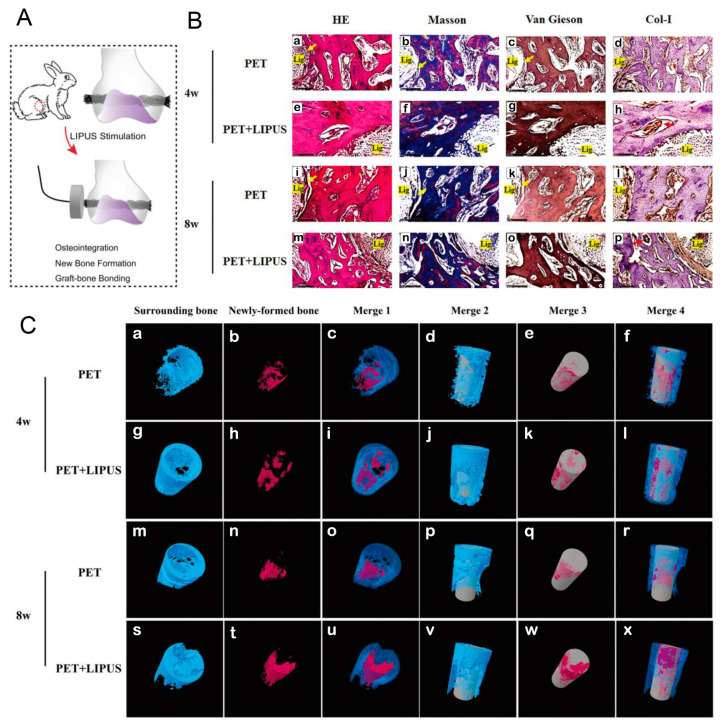
(**A**) Schematic illustration for the in vivo evaluation of LIPUS on the graft-bone healing of PET artificial ligaments. (**B**) HE, Masson, and van Gieson staining and Col-I immunohistochemical staining for the surrounding bone at 4 and 8 weeks postoperatively. Fibrous tissue (yellow arrow), Col-I (red arrow). (**C**) Micro-CT analysis of the surrounding bone and newly formed bone at 4 weeks and 8 weeks postoperatively. Blue, surrounding bone. Red, newly formed bone. Gray, PET graft. Reprinted with permission from Ref. [121]. Copyright 2022 American Orthopaedic Society for Sports Medicine.

Although artificial ligament grafts were a viable strategy for replacing autologous grafts, single artificial ligament grafts frequently resulted in poor integration. It was vital to design a strategy that was both effective and quickly useful for promoting graft-bone healing of artificial ligaments (Table 4). Liu et al. recently organized a study to investigate the effects of LIPUS on polyethylene terephthalate (PET) artificial ligament concerning cell behaviors in vitro and osseointegration in the extra-articular graft-bone healing model (Figure 7) [121]. LIPUS promoted the cell proliferation, adherence, and osteoblastic differentiation of MC3T3-E1 preosteoblasts seeded on PET sheets. Meanwhile, in vivo study of rabbits confirmed the promoting effect of LIPUS on bone formation, and enhanced effect on graft-bone healing, such as less fibrous tissue, narrower interface, direct contact and higher ultimate failure load [121].

LIPUS treatment appeared to accelerate bone–tendon interface healing after liposomal clodronate or liposomes injection. Additionally, liposomes + LIPUS exhibited significantly more fibrocartilage than liposomal clodronate + LIPUS. Biomechanical tests of mouse supraspinatus muscle–supraspinatus tendon–humerus structure was in line with the histological results [122].

LIPUS could facilitate the osteogenesis and microvascular formation of periodontal ligament stem cells [123,124]. Stimulated by LIPUS, autologous ADSC transplantation with fibrin can lead to superior bone–tendon healing quality in the patella–patellar tendon junctions when compared with LIPUS or ADSCs alone. Compared with other groups at postoperative 8 and 16 weeks, the LIPUS + ADSCs group showed more regeneration and maturity both in fibrocartilage layer and new bone histologically, and significantly higher ultimate failure load and stiffness biomechanically [125].

**Table 4:** LIPUS + biomaterials for bone–ligament/bone–tendon repair.

No		Biomaterials	Constituent	Evidence In Vitro				Evidence In Vivo				Ref.
				Cell	Prolif	Adhes	Osteog Differ	Animal	Osteoind	Bone Rem	Interf Heal	
1	Bone-Ligament	Polymer	PET	MC3T3-E1	+	+	+	Rabbit	+	/	+	[121]
2	Bone-Tendon	Lipidosome	Lipo clodro, Lipo,	/	/	/	/	Mouse	+	/	+	[122]
3	Bone-Tendon	Fibrin	ADSCs@Fib	/	/	/	/	Rabbit	+	+	+	[125]

Prolif: proliferation, Adhes: adhesion, Osteog Differ: osteogenic differentiation, Osteoind: osteoinduction, Bone Rem: bone remodeling, Interf heal: interface healing. PET: polyethylene terephthalate, Lipo clodro: liposomal clodronate, ADSCs@Fib: adipose-derived stromal cells loaded fibrin. +: Positive, /: not tested.

## 7. Nerve Repair

LIPUS affected the proliferation and myelinating activity of Schwann cells, in a both time- and duty ratio-dependent manner. Thus, LIPUS can be used to repair peripheral nerve injury and peripheral neuropathies [126,127]. The current knowledge about the influence of LIPUS on animal and human models revealed that LIPUS may have an impact on nerve regeneration and axonal alterations in the situation of carpal tunnel syndrome, transected nerve, dementia, and neurogenic erectile dysfunction [128,129]. Meanwhile, a growing number of studies are being conducted to investigate the function of LIPUS in materials-induced neuron regeneration (Table 5). LIPUS could accelerate autografting the sciatic nerve, and low-intensity US (250 mW/cm^2^) showed faster regeneration than higher intensity (500 and 750 mW/cm^2^) [130]. Notably, several studies reported that LIPUS could induce CGRP innervation, and then indirectly promote bone formation and spinal fusion [98,99,102,103].

Other studies focused on the potential accelerated effect of LIPUS on nerve conduit. L-ornithine was coated in the internal wall of PLGA conduits to promote cell adherence of the Schwann cells. In vitro, such conduit combined with LIPUS could promote cell proliferation of Schwann cells [131]. In vivo, the LIPUS stimulated the seeded Schwann cells to form regenerated nerves while inducing retarded axon regeneration in the silicone conduit [131,132].

**Figure 8:**
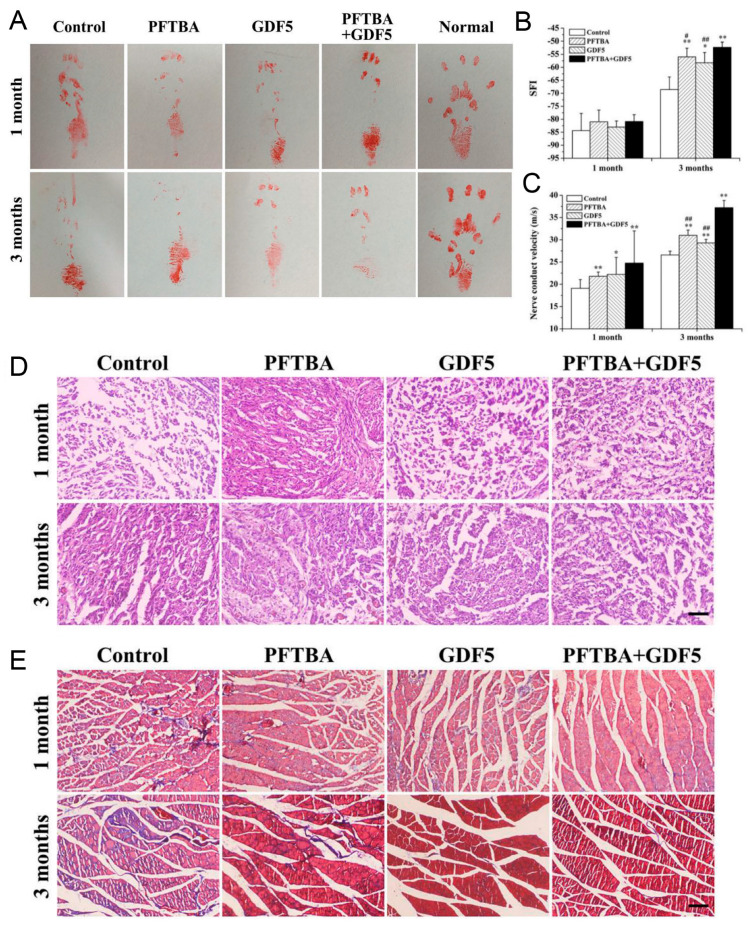
Promotion of LIPUS with allogeneic decellularized nerve conduit containing PFTBA and GDF5 on the repair of rat sciatic nerve injury. The footprint images (**A**), the SFI (**B**), and NCV (**C**) at 1 and 3 months post operation. * denotes *p* < 0.05 and ** denotes *p* < 0.01 vs. control group. # denotes *p* < 0.05 and ## denotes *p* < 0.01 vs. PFTBA+GDF5 group. Representative images of H&E staining (**D**) and Masson’s trichrome staining (**E**) on rat gastrocnemius muscle section at 1 and 3 months post operation. Reprinted with permission from Ref. [134]. Copyright 2019 Wiley.

After being blended with Matrigel solution, induced pluripotent stem cell-derived neural crest stem cells (iPSCs-NCSCs) were filled into the center of PLLA nanofiber nerve conduit, which acted as a scaffold in the rat transected sciatic nerve model. After LIPUS treatment, the neurophysiological parameters of the rat sciatic nerve were significantly improved. Staining of tissue sections revealed increased new blood vessels and neurofilaments, and increased expression of the neural marker Tuj1. Above all, the combination of LIPUS and iPSCs-NCSCs/PLLA promoted the regeneration and reconstruction of the rat sciatic nerve [133]. The activation of FAK-ERK1/2 signaling in iPSCs-NCSCs might contribute to the promotion effect of LIPUS on nerve regrowth. Based on this finding, this team fabricated an allogeneic decellularized nerve conduit containing iPSCs-NCSCs, perfluorotributylamine (PFTBA), and growth differentiation factor 5 (GDF5). The addition of PFTBA and GDF5 could provide an advantageous microenvironment for nerve regeneration, because PFTBA could supply enough oxygen and the addition of GDF5 could promote neural differentiation. This conduit showed rather good influence on the repair of rat transected sciatic nerves (Figure 8) [134].

Polycaprolactone/Pluronic F127 membrane (PCL/F127) was used to conduct a nerve guide conduit (NGC). The favorable permeability, hydrophilicity, and structural stability of PCL/F127 was good for the permeation of nutrients from the whole surroundings, whereas no nutrition could permeate the hydrophobic PLGA tube. Thus, this PCL/F127 conduit could favor nerve regeneration in the rat sciatic nerve defect model [135]. A further study used nerve growth factor (NGF) and LIPUS as double biophysical stimulation; by combining them, the NGF@PCL/F127 + LIPUS system could provide a synergetic effect on peripheral nerve repair, potentially for the repair of delayed and malfunctioned peripheral nerve [136].

**Table 5:** LIPUS + biomaterials for nerve repair.

No	Biomaterials	Constituent	Evidence In Vitro		Evidence In Vivo			Ref.
			Cell	Proliferation	Animal	Nerve Regeneration	Angiogenesis	
1	Composites	iPSCs-NCSCs@PLLA	/	/	Rat	+	+	[133]
2	Autograft	Autograft Nerve	/	/	Rat	+	/	[130]
3	Composites	iPSCs-NCSCs/PFTBA/GDF5A@ADNC	/	/	Rat	+	/	[134]
4	Composites	PCL/F127	/	/	Rat	+	/	[135]
5	Composites	NGF@PCL/F127	/	/	Rat	+	/	[136]
6	Composites	SC@PLGA	/	/	Rat	+	/	[132]
7	Composites	SC@PLGA	Schwann	+	Rat	+	/	[131]

iPSCs-NCSCs@PLLA: induced pluripotent stem cell-derived neural crest stem cells loaded poly(L-lactic acid), iPSCs-NCSCs/PFTBA/GDF5A@ADNC: allogeneic decellularized nerve conduit containing iPSCs-NCSCs, perfluorotributylamine (PFTBA), and growth differentiation factor 5 (GDF5), PCL/F127: Polycaprolactone/Pluronic F127 membrane, NGF@PCL/F127: nerve growth factor loaded PCL/F127 membrane, SC@PLGA: Schwann cells seeded poly(DL-lactic acid-co-glycolic acid) (PLGA). +: Positive, /: not tested.

## 8. LIPUS Parameters

When compared with other energies, ultrasound has a specific ability to deeply propagate within the human body. Furthermore, it is highly focused, making it an excellent source with high energy for clinical therapy. Based on the parameters and the type of tissue, there are both thermal and non-thermal effects after ultrasonic waves are penetrated into the body [137]. The US frequencies ranged from 1 to 15 MHz in medical application, with 1 MHz frequencies being used for therapeutic applications and 2.5 to 15 MHz frequencies for diagnostic procedures, depending on the depth and tissue type and the mechanics of mechanical wave propagation (Table 6) [38,138].

It is a challenging process to determine optimal LIPUS parameters because of the wide range of biomaterials that have been used. Thus, few studies have been conducted on LIPUS parameters that could interact with biomaterials on cells or in animal models. One study systematically investigated the acoustic excitation parameters, including intensity, frequency, duty cycle, and excitation duration. The most often used parameters were as follows: 30 mW/cm^2^ intensity, 1.5 MHz frequency, and 20% duty cycle, but no differences were observed for excitation durations of 1, 3, and 5 min [120]. After 5 days of LIPUS stimulation, the rBMSC cultured on tissue culture plates could express the highest ALP activity in the group of 30 mW/cm^2^ compared to the groups of 2 and 15 mW/cm^2^. However, the highest mineralization was observed in the group with 2 mW/cm^2^ after 17 days of LIPUS stimulation [139]. Zhou et al. proposed that the LIPUS intensity of 150 mW/cm^2^ from the tissue culture plate be used for further study using 3D printed scaffolds, since maximum proliferation of hBMSCs was observed at 150 mW/cm^2^ among five intensities (20, 50, 75, 150, and 300 mW/cm^2^) [52].

In the porous Ti6Al4V scaffolds, a LIPUS intensity of 30 mW/cm^2^ induced improved osteoblast differentiation when compared with 0, 10, and 60 mW/cm^2^ [57]. However, no significant differences on MC3T3-E1 cells cultured on Ti6Al4V scaffolds were noted between the 1 and 3.2 MHz frequencies, both in vitro and in vivo [58]. Mouse preosteoblast MC3T3-E1 cells encapsulated in type I collagen hydrogels induced higher COX-2 and PGE2 expression after LIPUS application in the 30 and 150 mW/cm^2^ group than in the 0 mW/cm^2^ group, and the expression of these two markers was even higher in the 30 mW/cm^2^ US intensity group than the 150 mW/cm^2^ group. However, COX-2 was an inducer of the expression of PGE2, both of them leading to the aggravation of tissue inflammation [70]. Hsu et al. determined that pulsed ultrasound was more effective in increasing ALP activity and cell proliferation than continuous ultrasound [56].

During the initial inflammatory phase of 2 postoperative weeks, the bone–tendon junction healed more quickly in the LIPUS group than the control group. The prominent relieving effect of LIPUS on local inflammation was validated by decreased mRNA expression of proinflammatory cytokines and increased anti-inflammatory cytokines of patella–patellar tendon complexes [140,141,142]. Additionally, further study showed that LIPUS initiated during postoperative week 1 had a more noticeable effect on bone–tendon healing compared to immediate postoperative healing and postoperative week 2 [143]. The ultrasonic intensity of 30 mW/cm^2^ was insufficient to promote axonal regeneration following nerve injury, as compared to the control and sham groups. It was recommended that the intensity of ultrasound should be adjusted to 200–300 mW/cm^2^ for clinical examinations [144]. Domenici et al. identified that a specific range of the exposure energy density (6.3–10.8 J/cm^2^) could modulate keratinocytes membrane trafficking with negligible biological damage [145]. After exposure to LIPUS (1 MHz, 65 mW/cm^2^) for 1 h, a significant transient deregulation of IL-6 expression and secretion was observed in keratinocytes. High LIPUS intensity could alter membrane permeability and further reduce cell viability [146].

Above all, LIPUS parameters varied with different biomaterials platforms, cell types, initiating exposure time, stimulate duration, and cell culturing techniques. Further systematic studies should be designed in order to confirm the optimal parameters for musculoskeletal tissue engineering. Generally, biomaterial-specific parameters or ranges of effective parameters might be promising solutions.

## 9. Conclusions and Future Perspective

LIPUS is a safe biophysiotherapy which is effective in the repair of musculoskeletal systems. However, there are several issues that need to be noted in the clinical translation of LIPUS with biomaterials on musculoskeletal tissue repair. Firstly, although the intensity of 30mW/cm^2^ is commonly used in most articles [147,148], the optimal LIPUS parameters may differ depending on the biological materials. However, there are few articles that have systematically explored material-specific LIPUS parameters. Secondly, to develop novel LIPUS-responsive biomaterials, a thorough understanding of the mechanisms of LIPUS is required because it could affect the regenerative microenvironments, including various cells, bioactive molecules, and implanted biomaterials. Even though the mechanism of LIPUS alone on cells has been studied in some papers, there are few studies focusing on the synergistic mechanisms between LIPUS and biomaterials. Thirdly, in addition to bone tissue engineering, researchers should focus on other challenging areas, such as LIPUS on tendon–bone healing, cartilage healing, and skeletal muscle regeneration [149,150].

Combining LIPUS and biomaterials that are approved for bone tissue engineering applications could result in an easier transition to the clinic. However, further exploration is needed in the area of integrating LIPUS with new types of biomaterials. Recently, an emerging trend could be observed of the application of metamaterials in bone repair. Metamaterials could be used to design a patient-specific implant for improving load transfer, or to design a printable tunable stiffness scaffold for bone healing [151,152]. However, there is still a lack of studies on the combination of LIPUS and metamaterials. In order to avoid redundant and meaningless work among peers, it is encouraged to report negative results in the research. LIPUS is recommended for various steps of musculoskeletal tissue engineering, such as the preparation of tissue engineering scaffolds, cell pretreatment, bone remodeling, and other processes. Soft tissues, including muscle, fat, and skin, cover the surface of the musculoskeletal system, but the thickness of the soft tissue varies among the different parts. It is a difficult challenge to convey LIPUS to deeper musculoskeletal tissue [23]. Thus, identifying the optimal LIPUS parameters for specific locations and materials will be crucial to improve the use of LIPUS for musculoskeletal tissue engineering.

Tissue regeneration is a dynamic process involving a bi-directional interaction between cells and the matrix surrounding them. This dynamic reciprocity may be enhanced by exogenous LIPUS stimuli, which could provide more signaling to the microenvironments of bone regeneration. Various changes within cells resulting from LIPUS may explain their alteration in response to local biological signals. In order to achieve new functionalities, specific or extensive strategies could be employed to exploit the physical effects of LIPUS on biomaterials, biological molecules, or cells. Further development also needs to be done on novel biomaterials with adaptable and responsive properties, whether they are stimulated by LIPUS or not.

## Figures and Tables

**Table 6:** Representative parameters for LIPUS + biomaterials.

Application	Intensity (mW/cm^2^)	Frequency (MHz)	Repetition Rate (kHz)	Pulse Burst (μs)	Duty Cycle (%)	Application (min/d)	Constituent	Equipment	Ref.
Bone regeneration	30	1.0	0.1	1000	NG	20	BaTiO_3_/TC4	Sonicator 740	[34]
Bone regeneration	30	1.5	1.0		20	20	BaTiO_3_/Ti6Al4V	Ronghai	[28]
Bone regeneration	100	3.0	NG	NG	50	10	cRGD-NBs	2776	[78]
Bone regeneration	300	1.0	NG	NG	NG	20	Collagen I	Agilent	[68]
Spinal fusion	30 ± 30%	1.5 ± 5%	1.0 ± 10%	200 ± 10%	20	20	DFDBA	Exogen	[97]
Spinal fusion	30	1.5	1.0	200	NG	20	AIBG	Exogen	[91]
Cartilage repair	60	1.5	1.0	NG	20	20	PBNPs	Osteotron IV	[119]
Cartilage repair	500	1.0	NG	NG	20	20	L-rapa	Intelect	[116]
Bone-Ligament or -Tendon repair	30 ± 5	1.5	1.0	200	NG	20	Liposomes	NG	[122]
Bone-Ligament or -Tendon repair	30	1.0	NG	NG	NG	20	PET	Osteotron IV	[121]
Nerve repair	250–750	1.0	NG	NG	20	5	Autograft Nerve	Customized device	[130]
Nerve repair	300–500	1.0	0.1	NG	20	45,214	iPSCs-NCSCs /PFTBA/GDF5A@ADNC	US10	[134]

NG: Not given, Ti6Al4V: titanium-6aluminum-4vanadium, cRGD-NBs: cyclic arginine-glycine-aspartic acid-modified nanobubbles, DFDBA: demineralized freeze-dried bone allograft, AIBG: autologous iliac bone graft, PBNPs: Prussian blue nanoparticles, L-rapa: liposome-encapsulated rapamycin, PET: polyethylene terephthalate, iPSCs-NCSCs/PFTBA/GDF5A@ADNC: allogeneic decellularized nerve conduit containing iPSCs-NCSCs.

## Data Availability

Not applicable.
